# Laryngeal electromyography in dysphonic patients with incomplete glottic closure

**DOI:** 10.5935/1808-8694.20120026

**Published:** 2015-10-20

**Authors:** Noemi Grigoletto De Biase, Gustavo Polacow Korn, Grazzia Gugliemino, Paulo Pontes

**Affiliations:** Senior Associate Professor at the Federal University of São Paulo/Paulista Medical School; PhD in Sciences by the Federal University of São Paulo/Paulista Medical School; Fellow in Laryngology by the Federal University of São Paulo/Paulista Medical School; Professor of the Department of Otorhinolaryngology and Head and Neck Surgery at the Fellow in Laryngology by the Federal University of São Paulo/Paulista Medical School (Chairman of IFOS (International Federation of ORL Societies))

**Keywords:** dysphonia, electromyography, laryngeal muscles, larynx

## Abstract

The lack of specificity in laryngoscopical examination requires that the diagnosis of superior laryngeal and recurrent laryngeal nerve involvement be carried out with the aid of electromyography.

**Objective:**

This study aims to assess the electrophysiological function of the superior and inferior laryngeal nerves by measuring the electrical activity of the muscles they innervate in dysphonic patients with incomplete closure of the vocal folds during phonation.

**Method:**

Thirty-nine patients with incomplete glottic closure were enrolled in a prospective study and had their cricothyroid, thyroarytenoid, and lateral cricoarytenoid muscles examined bilaterally through electromyography. Insertion activity, electrical activity at rest (fibrillation, positive wave and fasciculation) and during muscle voluntary contraction (recruitment, amplitude, potential length and latency between electrical activity and phonation) were measured.

**Results:**

No altered test results were observed for parameters insertion activity and electrical activity at rest. None of the patients had recruitment dysfunction. The mean electrical potential amplitude values were within normal range for the tested muscles, as were potential durations and latency times between the onset of electrical activity and phonation.

**Conclusion:**

No signs of denervation were seen in the thyroarytenoid, cricothyroid, and lateral cricoarytenoid muscles of the studied patients.

## INTRODUCTION

The clinical relevance of electromyography (EMG) has been recognized in recent years^1^, ^2^, ^3^, ^4^, ^5^, ^6^. EMG has become an important method in assessing laryngeal diseases, more specifically in patients with involved vocal fold mobility. The technical difficulty that characterizes this test, however, has limited its use. It is particularly useful in diagnosing inferior motor neuron disorders, differentiating fixation paralyses, and in locating lesions and establishing prog-nosis^1^, ^2^, ^3^, ^7^, ^8^, ^9^, ^10^, ^11^, ^12^, ^13^, ^14^, ^15^, ^16^, ^17^, ^18^, ^19^, ^20^, ^21^, ^22^, ^23^, ^24^, ^25^, as it is the most objective method to assess muscle activity^14^.

Inferior laryngeal nerve unilateral involvement can be diagnosed by analyzing the patient's phonation and the observation of varying degrees of breathiness. When the superior laryngeal nerve is involved, subjects have difficulty adjusting voice loudness and singing.

Recurrent laryngeal nerve dysfunction is also easily verified through laryngoscopy, as vocal fold mobility is impaired in these cases. The alterations consequent to the loss of function of the cricothyroid muscle - innervated by the superior laryngeal nerve - are not evident, and thus hamper diagnostic efforts even with the aid of videolaryn-goscopy^2^. The most common alterations are amplitude and mucosal wave phase asymmetries, delayed abduction and adduction of the involved vocal fold, and glottic clefts^5^, all of which are non-specific findings. Therefore, isolated cases of superior laryngeal nerve paralysis or paresis with preserved vocal fold mobility - an occurrence of remarkable relevance for voice professionals - are hard to diagnose. While disorders involving the recurrent laryngeal nerve and the lateral cricoarytenoid (LCA) muscle are more easily diagnosed in clinical examination by the absence of rotation of the arytenoid cartilage and fixed position of the vocal folds, the isolated involvement of branches of the thyroarytenoid muscle introduces only a few disturbances. Examination with a laryngoscope reveals little evidence of involvement, such as non-specific spindle-shaped clefts^26^, ^27^.

Superior laryngeal nerve injuries can be diagnosed through EMG of the cricothyroid muscle^4^, ^7^, ^11^, ^13^, ^14^, ^15^, ^16^. The examination of the muscles innervated by the recurrent laryngeal nerve requires accurate knowledge on the anatomy of the larynx and experience with EMG. EMG of the thyroarytenoid and LCA muscles assesses the activity of the branches of the recurrent laryngeal nerve in the main larynx adductors, and rules out the need to examine the bilaterally innervated interarytenoid muscles.

This study aimed to assess the electrophysiological function of the recurrent laryngeal and superior laryngeal nerves by looking into the electrical activity of the muscles they innervate in patients with phonation disorders and incomplete coaptation of the vocal folds during phonation as seen in video laryngoscopy.

## METHOD

This project was approved by the Research Ethics Committee at the Federal University of São Paulo – Paulista Medical School (permit n^o^ 0122/04).

This cross-sectional prospective study enrolled individuals seen between October 2005 and March 2007 with complaints of dysphonia and incomplete closure of the vocal folds observed in video laryngoscopy. The subjects underwent laryngeal examination by EMG. A control group was not required, as the parameters have been clearly established.

Enrollment criteria: subjects had to be 18 or older, present complaints of dysphonia and incomplete glottic closure as observed in video laryngoscopy, except for presence of a posterior triangular cleft in females.

Exclusion criteria: lack of vocal fold mobility as seen in laryngoscopy, presence of lesions or degenerative disease in the vocal folds.

Sample characterization: 39 subjects, 16 males and 23 females, aged between 18 and 80 years, with a mean age of 51.

### Procedure

All patients underwent ENT examination including video laryngostroboscopy or fibrolaryngoscopy with stro-boscopy. The subjects were examined through EMG after ENT examination.

Video laryngostroboscopy was carried out with the patients under topical anesthesia with 10% lidocaine; subjects were asked to utter vowel ‘é ' as closely as possible to the way they would in spontaneous phonation. EMG was performed by the same examiner for every patient. Patients were asked to sit and place their heads against a slightly tilted head rest, at an angle of approximately 30 degrees. Before the test was done the patients had their skin cleaned with 70% ethanol. EMG examination was done using a Nihon Kohden® Neuropack device with a 10-kHz low pass filter and a 10-kHz high pass band filter with two channels. The first channel was used for the needle electrode recording muscle electrical activity. The second channel was used to capture the sounds uttered by the patients into a microphone positioned 10 cm away from their mouths. The ground wire was placed close to the patients' sternum. Time was measured in units of 10 ms and with sensitivity ranging from 100 to 500 µV. All exams were carried out using a concentric bipolar needle electrode.

Examination was conducted as follows, starting on the left side, then moving on to the right side, in accordance with the technique:

1. Palpation of the neck and identification of the anatomic landmarks to position the needle: thyroid and cricothyroid cartilages, cricothyroid membrane.
2. Introduction of the bipolar concentric electrode by percutaneously puncturing the skin over the cricothyroid membrane approximately 0.5 cm to 1.0 cm away from the median sagittal line, between the upper border of the cricoid cartilage and the lower border of the thyroid cartilage, all the way to the cricothyroid muscle at a depth of 0.5 cm to 1.0 cm. The muscle is identified by the presence of audible potentials verified on a computer screen as the patients are asked to produce high frequency sounds.
3. Percutaneous introduction of the needle through the cricothyroid membrane, driving the needle at an angle of 30 to 45 degrees in the lateral and superior directions depending on the subject's neck anatomy, until the thyroarytenoid muscle is reached, at a depth of approximately two centimeters.
4. Introduction of the needle by percutaneously puncturing the skin over the cricothyroid membrane in the lateral portion of the larynx, in the area close to the end of the thyroid cartilage, until the LCA muscle is reached.

EMG was performed to assess spontaneous and voluntary muscle activity; the data sets gathered from the examinations were recorded onto the device's hard drive for further analysis. The following parameters were recorded for each studied muscle: electrical activity at rest, i.e., during normal breathing; electrical activity as patients uttered vowel ‘e' at their usual intensity and frequency levels as the thyroarytenoid and LCA muscles were tested; and electrical activity as patients produced high frequency sounds to assess the cricothyroid muscle. The analysis of EMG records included assessment of insertion activity levels, spontaneous activity levels at rest, and activity levels during phonation.

Insertion activity is the electrical response obtained as the needle is driven into the muscle, probably generated by the mechanical process of the needle penetrating the muscle tissue. This parameter was categorized as normal, reduced, or increased.

Spontaneous activity is the electrical response generated spontaneously while the muscle is at rest and the needle is kept still. This parameter was categorized as absent - usually observed in normal muscle tissues - or present. Presence of spontaneous activity was characterized by the identification of fibrillations, positive waves, fasciculation, and high frequency complexes. Fibrillations are low amplitude potentials (10 to 200µV) of short duration (1 to 2 ms). Positive waves present a positive component in amplitudes ranging between 20 and 200 µV and a negative minimum component. Fasciculations are discharges with varied morphology arising from one or part of a motor unit High frequency discharges are bizarre discharges of variable duration identified mainly by sound.

The analysis of voluntary activity included the assessment of the following parameters:

1. Motor unit recruitment patterns were categorized as normal or with rarefaction, based on the presence of peaks leading to the disappearance of the baseline, at an acquisition rate of 100 ms/cm.
2. Wave duration is the time between the point at which the wave starts from the baseline and its return. This parameter was measured using the software on the EMG device. A window was manually configured to identify the beginning and the end of the wave, and the software calculated its duration. The durations of two clearly defined waves were measured to allow the calculation of the mean duration value. Mean values were categorized as normal (3-7 ms)^14^, ^28^, ^29^, ^30^, increased, or reduced.
3. Motor unit action potential (MUAP) amplitude is the measurement obtained between the most negative and the most positive peaks found for the wave. It was measured using a software that used the data stored on a text file to find the mean values of the acquired potentials by calculating the square roots of the potentials to the square of each wave component, thus covering negative and positive values. In the measurement of the mean amplitude values, the minimum and maximum times of analysis considered were 1 and 2 seconds respectively. Mean amplitudes were categorized as normal (188-1485 µV)^29^, increased, or reduced.
4. During the examination of the thyroarytenoid muscle, one of the voluntary activity values was obtained by pushing the device's button that quantifies the intensity of the sound generated by electrical activity at the minimum volume level, i.e., without sound. This file was used to measure the latency between the onset of electrical activity acquired on channel 1 and the appearance of the sound uttered by the subject acquired on the microphone connected to channel 2 on the device. Latency times were measured from the onset of increased electrical activity from the baseline on channel 1 to the onset of activity on the voice channel (channel 2). Latency times were deemed normal when equal to or under 408 ms^29^ or increased when above the threshold.

Based on the findings related to electrical activity at rest and during voluntary activation, EMG examination of each laryngeal muscle may indicate the following:

1. Absence of electrophysiological alterations: normal insertion activity, absence of spontaneous activity, normal amplitude and duration, normal latency time.
2. Neurogenic lesion: normal, increased, or reduced insertion activity; normal latency time. Acute involvement was characterized when fibrillation-type spontaneous activity or positive wave and rarefaction were found. The presence of rarefaction and absence of spontaneous activity were interpreted as neuropraxia. Chronic involvement was identified when fibrillation-type spontaneous activity or positive waves were not found, fasciculation or high frequency discharge was present or absent, and rarefaction and waves with increased duration and amplitude were found. The presence of rarefaction and absence of spontaneous activity, along with normal wave duration and amplitude, was characterized as chronic neurogenic injury with central involvement.
3. Myopathic lesion: normal insertion activity, reduced MUAP amplitude and duration.
4. Inconclusive: findings did not fit any of the categories described above.

The electrical activity of the thyroarytenoid and LCA muscles was analyzed to assess recurrent laryngeal nerve function, whereas the cricothyroid was used to look into the superior laryngeal nerve bilaterally. The thyroarytenoid and the LCA are innervated by the recurrent laryngeal nerve, but both must be analyzed as patients may have selective cases of paresis - e.g.: thyroarytenoid paresis -which are difficult to characterize through laryngoscopy and require EMG examination to confirm the diagnosis^19^.

The subjects were provided with clarification on the research and on the tests they signed up to undergo and signed a free informed consent form.

## RESULTS

Nine of the 39 patients submitted to EMG of the larynx did not have their LCA muscles examined due to technical hazards in locating their muscles or because they asked for a shorter course of tests. The thyroarytenoid muscle was normal in all such patients. In three patients the cricothyroid muscle was not found - two on the right side and one on the left side. The difficulty identifying the three cricothyroid muscles may be explained by the approached picked to perform the examination: the same puncture was used to look at the cricothyroid and thyroarytenoid muscles, and the puncture may have been performed medially to the cricothyroid. In one patient, only the cricothyroid muscles were examined and in another patient only the muscles on the left side were observed. All other patients (25) had all the described muscles examined.

The mean latencies between electrophysiological activity and start of phonation in the right and left thyroarytenoid muscles was 230.9 ms and 194.4 ms respectively; MUAP durations in the left (LCT) and right (RCT) cricothyroid muscles, left (LTA) and right (RTA) thyroarytenoid muscles, left (LLCA) and right (RLCA) lateral cricoarytenoid muscles were 4.1 ms, 3.5 ms, 4.7 ms, 4.6 ms, 4.8 ms, and 5.0 ms respectively.

The mean MUAP amplitude on muscles LCT, RCT, LTA, RTA, LLCA, and RLCA were 626.7 µV, 703.9 µV, 688.8 µV, 725.7 µV, 662.0 µV, and 679.9 µV respectively.

The analysis of EMG results shows that wave morphology was normal and none had pathologic spontaneous activity or rarefaction. MUAP duration was found to be within normal range, as were latencies between electrophysiological activation and phonation (Table 1). Mean MUAP amplitude was within normal range for all tested muscles (Table 2).

**Table 1:** Latency between electrophysiological activity and onset of phonation (ms) and MUAP duration in the examined muscles (ms).

Patient	Latency				Duration			
	LTA	RTA	LCT	RCT	LTA	RTA	LLCA	RLCA
1	404	350	4.7	3.8	4.2	5.3	-	-
2	274	NI	4.2	3.7	4.3	5.1	5.7	4.6
3	145	195	3.7	3.2	4.7	4.5	-	-
4	NI	NI	2.9	5.3	4.2	4.6	-	-
5	204	250	2.9	3.5	3.7	4.2	3.3	3.6
6	178	78	4.9	5.7	4.1	3.3	5.3	6.5
7	190	178	2.6	4.7	5.3	3.4	6.8	6.1
8	125	115	2.5	4.2	3.4	3.3	4.6	2.8
9	274	129	4.0	4.2	3.9	4.3	5.0	4.6
10	321	418	3.5	3.8	5.2	4.5	-	-
11	NI	NI	4.8	4.2	3.7	4.3	5.2	5.7
12	123	42	3.5	3.3	4.3	2.7	3.2	3.5
13	236	NI	2.7	3.2	3.3	3.4	-	-
14	197	NI	2.7	-	5.6	-	5.7	-
15	379	261	4.4	4.9	5.7	4.8	2.8	4.7
16	123	144	3.9	3.7	3.3	5.2	-	-
17	311	253	2.4	5.2	4.2	3.1	4.7	4.8
18	231	140	4.7	4.4	2.7	5.0	-	-
19	220	240	5.9	6.5	5.9	5.8	3.1	3.7
20	350	325	3.3	3.8	3.4	5.4	5.9	6.7
21	275	120	3.6	3.8	3.2	4.8	4.6	4.8
22	196	239	5.6	6.6	5.9	5.7	5.4	5.5
23	63	91	3.9	3.6	5.4	4.8	5.8	5.9
24	NI	NI	5.2	3.5	5.8	4.6	3.3	5.3
25	NI	NI	3.7	4.2	-	-	-	-
26	197	78	5.5	3.8	6.5	5.2	-	-
27	312	335	4.9	3.4	4.7	3.8	4.7	5.3
28	95	233	5.9	4.8	4.9	4.2	5.2	4.2
29	179	124	4.9	3.5	4.6	3.9	4.6	5.3
30	215	NI	6.5	4.2	4.0	6.4	5.9	4.7
31	153	115	3.0	-	4.7	4.0	4.1	6.1
32	417	278	4.3	5.4	5.7	4.7	6.8	5.3
33	279	82	3.9	3.3	4.4	5.8	5.7	4.5
34	346	124	4.2	3.2	5.0	4.3	4.6	5.7
35	225	NI	3.0	3.0	4.7	4.0	5.3	5.2
36	283	248	5.9	6.4	5.6	5.7	3.4	4.8
37	154	226	2.7	3.2	5.1	2.9	4.5	4.8
38	NI	158	4.6	5.1	6.9	5.7	2.8	5.3
39	176	264	5.1	3.8	6.1	6.8	5.7	4.4
mean	230.9	194.4	4.1	3.5	4.7	4.6	4.8	5.0
median	217.5	186.5	4.0	3.8	4.7	4.6	4.9	4.8
minimum	63	42	2.4	3.0	2.7	2.7	2.8	2.8
maximum	417	418	6.5	6.6	6.9	6.8	6.8	6.7

muscles: left thyroarytenoid (LTA); right thyroarytenoid (RTA); left cricothyroid (LCT); right cricothyroid (RCT); left lateral cricoarytenoid (LLCA); right lateral cricoarytenoid (RLCA); NI: not included.

**Table 2:** Mean MUAP amplitude (µV).

Patient	Mean MUAP amplitude					
	LCT	RCT	LTA	RTA	LLCA	RLCA
1	491.2	948.7	1152.1	1059.8	-	-
2	416.3	531.1	996.5	1104.8	798.3	854.7
3	168.0	425.6	1399.2	547.9	-	-
4	1146.1	402.4	599.7	363.5	-	-
5	474.2	410.0	795.4	331.7	543.7	438.6
6	795.8	632.7	674.8	685.4	411.3	1144.4
7	355.4	739.6	626.7	475.4	845.0	600.1
8	765.4	586.1	1319.9	937.1	677.5	730.0
9	291.4	896.9	548.3	799.2	509.5	691.7
10	391.3	620.7	612.8	505.3	-	-
11	573.8	699.3	575.7	546.6	361.3	377.5
12	668.2	367.5	628.2	400.5	354.2	367.2
13	766.5	888.9	753.7	547.9	-	-
14	1128.8	NI	419.3	NI	1309.9	NI
15	225.2	419.2	326.5	600.8	409.1	548.9
16	NI	1276.8	864.3	1294.9	-	-
17	789.1	872.9	602.5	827.6	454.4	841.7
18	628.9	605.3	603.5	595.1	-	-
19	713.6	459.1	744.3	467.3	1203.5	589.0
20	376.6	566.4	373.1	809.9	487.9	568.9
21	667.7	413.3	415.3	500.5	787.5	865.8
22	385.7	907.5	513.4	831.6	493.9	376.5
23	385.1	767.3	410.5	577.5	598.0	394.1
24	958.5	453.4	347.6	491.1	453.3	476.9
25	626.1	636.7	NI	NI	-	-
26	500.7	1142.5	682.9	638.6	-	-
27	547.5	424.0	356.5	580.0	1484.7	1013.5
28	762.0	1370.0	534.7	609.1	657.3	754.4
29	515.4	1272.7	795.1	1430.6	456.7	643.2
30	1143.1	759.1	1399.2	1016.6	857.6	753.0
31	933.9	NI	298.1	421.4	487.4	435.9
32	607.9	537.1	851.4	788.5	787.1	653.3
33	197.7	498.2	789.3	1281.0	710.9	1432.6
34	758.3	678.9	546.7	678.9	489.5	578.9
35	978.4	1105.5	678.1	864.8	534.9	650.2
36	537.8	789.2	567.7	675.3	748.2	890.2
37	764.8	598.3	1120.5	978.9	734.7	756.8
38	890.4	796.7	540.0	749.0	557.8	638.9
39	489.5	546.3	758.3	837.5	655.8	534.8
mean	626.7	703.9	688.8	725.7	662.0	679.9
median	617.0	632.7	619.8	675.3	577.9	641.1
minimum	168.0	367.5	298.1	331.7	354.2	534.8
maximum	1146.1	1370	1399.2	1430.6	1484.7	1432.6

muscles: left thyroarytenoid (LTA); right thyroarytenoid (RTA); left cricothyroid (LCT); right cricothyroid (RCT); left lateral cricoarytenoid (LLCA); right lateral cricoarytenoid (RLCA). NI: not included.

Therefore, no functional electrophysiological alterations were seen in the superior and recurrent laryngeal nerves of the patients with incomplete glottic closure and dysphonia.

## DISCUSSION

EMG is an extremely useful tool in diagnosing neurogenic diseases, diseases of the neuromuscular junction, and myopathies, and has been broadly applied in large skeletal muscles^1^, ^3^, ^9^, ^10^, ^11^, ^12^, ^13^, ^14^, ^18^, ^19^, ^20^. In patients suspected for neuromuscular junction involvement and myopathies, EMG can be done in accessible muscles such as the levator palpebrae superioris, the orbicularis oculi, the biceps, and the quadriceps.

In laryngeal mobility disorders, EMG allows the examiner to locate the lesion, define which nerves were involved, and draw out prognostic parameters^1^, ^3^, ^5^, ^9^, ^10^, ^11^, ^12^, ^13^, ^14^, ^18^, ^20^, ^21^, ^22^, ^23^.

EMG of the larynx has its particularities, and requires knowledge on the anatomy of the structures of the larynx, more specifically the location of the intrinsic muscles and the pathophysiology of the diseases that may hamper the function of the larynx as the protective barrier for the lower airways and the organ responsible for phonation^4^, ^7^.

The intrinsic laryngeal muscles are small and located close to each other and other muscles in the neck area, as the extrinsic laryngeal muscles. The larynx has antagonist muscles which, by means of the motion of the arytenoids - mainly its rotation on the cricoid cartilage, lead to the abduction of the vocal folds and, consequently, the free inflow and outflow of air, or adduction, during swallowing or phonation.

The innervation of the antagonist muscles is done by the recurrent laryngeal nerve, a branch of the vagus nerve, whose motor neurons are located in the nucleus ambiguus, bilaterally in the brainstem. The recurrent laryngeal nerve stretches throughout the neck, and is thus susceptible to trauma and compressive injuries, and lesions subsequent to thyroidectomy^9^, ^10^, ^13^, ^31^.

Laryngeal mobility disorders bear significant clinical consequences and repercussions upon phonation when occurring unilaterally, and upon breathing when involvement is bilateral.

The vagus nerve, through its superior laryngeal branch, innervates the cricothyroid muscle which, by the forward rocking of the thyroid cartilage and backward rocking of the cricoid cartilage, elongates and tenses the vocal folds, making it possible for people to utter high frequency sounds which are critical in singing. Injuries to the superior laryngeal nerve - paralysis or paresis - do not lead to laryngeal immobility, but only to indirect signs suggestive of involvement. The diagnosis of muscle paralysis is done through EMG. Thus, in order to assess the recurrent and superior laryngeal nerves, the electrophysiological activity of at least two laryngeal intrinsic muscles should be considered: the cricothyroid, evaluated in high frequency utterances, and the thyroarytenoid through phonation at low and high frequencies.

The recurrent laryngeal nerve branches out after it penetrates the larynx, and usually its first branch moves toward the posterior cricoarytenoid abductor muscle. The second branch reaches the interarytenoid muscle's both sides, and follows on to the LCA to finally reach the terminal branch of the thyroarytenoid^31^. Albeit rate, selective paresis of its branches may occur and diagnosis can only be performed using EMG^24^. In this case, knowledge of clinical alterations may require the assessment of other intrinsic muscles which are harder to access and, thus, are not routinely examined.

Incomplete closure of the vocal folds leads to dysphonia, varying degrees of breathiness, reduced intensity, compromised voice efficiency, and exhaustion when speaking. Some alterations in the configuration of the larynx during phonation may be consequent, among other factors, to neurological involvement. Thus, spindle-shaped clefts have been related to paresis and paralysis of the thyroarytenoid and cricothyroid muscles^23^. EMG allows for the diagnosis of these diseases and, thus, provides information to define the best course of therapy. Accurate diagnosis of dysphonia, a key component in defining the best therapeutical approach and prognostic factors, is not always possible through laryngoscopy. This paper aimed to assess electrophysiological activity in the cricothyroid, thyroarytenoid, and LCA muscles and analyze the function of the recurrent and superior laryngeal nerves in individuals with glottic cleft. All analyzed parameters were found to be within normal range as described in the literature^23^, ^25^, ^28^, ^29^, ^30^. Normal values were obtained from the literature as, for ethical reasons, it is virtually impossible to perform EMGs in healthy individuals on a control group^14^, ^28^, ^29^, ^30^. The muscles tested in all patients were found to be normal, therefore ruling out the possibility of neural involvement^23^, ^28^, ^29^, ^30^ in the pathogenesis of incomplete glottic closure.

This fact may indicate that the way EMG is performed and the parameters currently considered may be insufficient to identify the electrophysiological alterations possibly in place or to indicate that clefts are present for other reasons, such as structural tissue modifications probably arising from the lamina propria. Or yet, to relate them to muscle alterations that do not lead to changes in muscle electric activity. Another hypothesis to be considered is the low prevalence of diseases in the superior laryngeal nerve, which could explain the absence of patients with such type of involvement in our sample. Koufman et al.^32^ performed larynx EMG in patients with spindle-shaped clefts and found signs of involvement in the superior laryngeal nerves of 16% of their patients. However, other findings were observed through laryngoscopy, such as arytenoid rotation and hypermobility, which resulted in indication to perform EMG. Our study was different in this regard to that done by Koufman et al., as in our sample we had no patients with altered laryngeal mobility. Therefore, more studies are required to shed light on the factors connected to glottic insufficiency.

## CONCLUSION

Electrophysiological assessment of the superior and recurrent laryngeal nerves failed to detect disorders in patients with dysphonia and incomplete glottic closure seen through video laryngostroboscopy.
