# Erratum of “Laryngeal electromyography in dysphonic patients with incomplete glottic closure”^☆^

**DOI:** 10.1016/j.bjorl.2018.10.001

**Published:** 2018-11-08

**Authors:** Noemi Grigoletto de Biase, Gustavo Polacow Korn, Grazzia Guglielmino, Paulo Pontes

**Affiliations:** a Livre-Docente pela Universidade Federal de São Paulo/Escola Paulista de Medicina; b Doutor em Ciências pela Universidade Federal de São Paulo/Escola Paulista de Medicina; c Fellow em Laringologia pela Universidade Federal de São Paulo/Escola Paulista de Medicina; d Professor Titular pelo Departamento de Otorrinolaringologia e Cirurgia de Cabeça e Pescoço da Universidade Federal de São Paulo/Escola Paulista de Medicina (Presidente da IFOS (International Federation of ORL Societies)), Universidade Federal de São Paulo/Escola Paulista de Medicina

In the article **“Laryngeal electromyography in dysphonic patients with incomplete glottic closure”** published in Braz J Otorhinolaryngol. 2012;78(6):7–14, where it reads Grazzia Gugliemino, it should read Grazzia Guglielmino.

